# Pooled-analysis of tadalafil and tamsulosin for ureteral calculi

**DOI:** 10.3389/fphar.2024.1351312

**Published:** 2024-05-30

**Authors:** Fengze Sun, Hongquan Liu, Gang Wu, Ming Liu, Shangjing Liu, Lin Wang, Qingsong Zou, Yuanshan Cui, Jitao Wu

**Affiliations:** ^1^ Department of Urology, Yantai Yuhuangding Hospital, Qingdao University, Yantai, Shandong, China; ^2^ The Second Clinical Medical College, Binzhou Medical University, Yantai, Shandong, China

**Keywords:** tadalafil, tamsulosin, distal ureteral calculi, meta-analysis, prospective randomized trials

## Abstract

**Objective:**

Urolithiasis is a common urological diseases and affects the daily life of patients. Medical expulsive therapy has become acceptable for many parents. We conducted a meta-analysis to determine the efficacy and safety of tadalafil compared with tamsulosin for treating distal ureteral stones less than 10 mm in length.

**Methods:**

Related studies were identified via searches of the PubMed, Embase, and Cochrane Library databases. All the articles that described the use of tadalafil and tamsulosin for treating distal ureteral stones were collected.

**Results:**

A total of 14 studies were included in our meta-analysis. Our results revealed that tadalafil enhanced expulsion rate [odds ratio (OR) = 0.68, 95% confidence interval (CI): 0.47 to 0.98, *p* = 0.04]; reduced expulsion time [mean difference (MD) = 1.22, 95% CI (0.13, 2.30), *p* = 0.03]; lowered analgesia use [MD = 38.66, 95% CI (7.56, 69.77), *p* = 0.01] and hospital visits [MD = 0.14, 95% CI (0.06, 0.22), *p* = 0.0006]. According to our subgroup analysis, either tadalafil 5 mg or 10 mg did not promote expulsion rate and accelerate expulsion time compared with tamsulosin. But patients receiving 5 mg tadalafil decreased analgesia usage [MD = 101.04, 95% CI (67.56, 134.01), *p* < 0.00001].

**Conclusion:**

Compared with tamsulosin, tadalafil demonstrates a higher expulsion rate and less expulsion time for patients with distal ureteral stones less than 10 mm with a favorable safety profile.

## 1 Introduction

Urolithiasis is a common urologic disease that affects patients’ health and places a heavy burden on the healthcare system (Shokeir et al., 2016). The occurrence of urolithiasis is primarily associated with race, age, sex, and region (Manglaviti et al., 2011). Among urolithiasis, the incidence of ureteral calculi is approximately 20%, with distal ureteral calculi accounting for 68% of these calculi (Hollingsworth et al., 2006; Bai et al., 2017).

The treatments of distal ureteral calculi are medical expulsive therapy (MET) and surgery, which is chosen according to stone size and stone location. Generally, ureteral stones larger than 10 mm in diameter are less likely to pass spontaneously, leading to hydronephrosis and secondary infection. Patients with large stones are treated with extracorporeal shock wave lithotripsy (ESWL), ureteroscopic lithotripsy (URSL), percutaneous nephrolithotripsy (PCNL), and laparoscopic ureterolithotomy (LU) (Elmansy and Lingeman, 2016; Kadyan et al., 2016; Tugcu et al., 2016; Rukin et al., 2017). According to the American Urological Association (AUA) guidelines, the exclusion rate automatically for stones with diameters less than 5 mm was 68%. Whereas for larger stones (6–10 mm), it was reduced to 48%. However, the MET could significantly increase the expulsion rate.

There is increasing evidence that patients with ureteral calculi benefit from alpha adrenoreceptor antagonists and phosphodiesterase type 5 inhibitors (PDE5-Is) (Özsoy et al., 2016). The main mechanism is that the alpha-1 receptor is distributed in the distal third of ureteric smooth muscle, and alpha blockers suppress basal smooth muscle tone while preserving tonic propulsive contractions to promote stone discharge (Yilmaz et al., 2005). The main mechanism of action of PDE5-Is differs from that of alpha blockers. PDE5-Is relax the ureter and dilate the lumen of the ureter to allow stones to pass spontaneously through the smooth muscle nitric oxide/cyclic guanosine monophosphate signaling pathway.

Several studies have shown that alpha blockers, including tamsulosin, can be used as primary drugs for the treatment of lower urinary tract stones (Parsons et al., 2007; Lu et al., 2012). The PDE5-Is tadalafil is commonly used to treat sexual dysfunction and lower urinary tract symptoms, and it is also adopted for distal ureteral stones (Bai et al., 2017; Zhou et al., 2019). Although some studies have compared the safety and efficacy of tamsulosin and tadalafil for distal ureteral stone treatment (Bai et al., 2017; Montes Cardona and García-Perdomo, 2017; Li et al., 2019), the results could be limited by the small size of included studies.

This meta-analysis aimed to compare the safety and efficacy of tamsulosin and tadalafil for distal ureteral stones smaller than 10 mm by incorporating updated evidence.

## 2 Method

### 2.1 Search strategy

The PRISMA guidelines were applied in our study (Page et al., 2021) (Supplementary Table S1). Prospective randomized clinical trials investigating the efficacy of tamsulosin and tadalafil in treating distal ureteral stones were identified by three authors in the PubMed (until April 2024), Embase (until April 2024), and Cochrane Library databases (until April 2024). The search terms included: tamsulosin, tadalafil, stone, calculi, distal ureter, lower ureter, and randomized. The authors also reviewed these studies to confirm their availability and identify any additional relevant articles.

### 2.2 Inclusion criteria

The inclusion criteria for this study were: 1) comparing tamsulosin and tadalafil for the distal ureteral stone treatment; 2) inclusion of prospective randomized clinical trial; 3) distal ureteral stone size less than 10 mm; and 4) provision of accurate data, including stone evaluation indices like expulsion rate and time. The details of the inclusion criteria are shown in Supplementary Table S2. Letters, comments, reviews, and qualitative studies were excluded. If multiple experiments involved the same participant group by different researcher, all studies were included.

### 2.3 Quality assessment

The quality of all included studies was assessed following the guidelines outlined in the Cochrane Handbook (Vader, 1998). Each studies was categorized based on the quality assessment criteria as follws: (+) low risk of bias; (?) moderate risk of bias; (−) high risk of bias. The guidelines outlined in The Cochrane Handbook for Systematic Reviews of Interventions V.5.4.0 were also employed to access each included study (Cumpston et al., 2019). All discrepancies in classification among the authors were resolved through discussion.

### 2.4 Data extraction

Various valuable data was collected from all included studies, encompassing: a) study type, b) first author’s name, c) publication date, d) sample size, e) eligibility criteria, and exclusion criteria, interventions, follow-up period, and study date; f) expulsion rate, expulsion time, analgesic use, colic episodes, hospital visit; and g) side effects including headache, backache, dizziness, abnormal ejaculation, gastritis, and orthostatic hypotension.

### 2.5 Statistical and meta-analysis

All data from the included studies underwent statistical analysis using Review Manager software (RevMan, version 5.3.0; Cochrane Collaboration) (Cumpston et al., 2019). Continuous data were analyzed by mean difference (MD), while dichotomous data were evaluated by odds ratios (ORs) with 95% confidence intervals (95% CIs) (DerSimonian and Laird, 2015). A heterogeneity test was conducted due to variations in patient populations. If the *p*-value was less than 0.05, the enrolled studies were considered heterogeneous. Estimates were conducted with the random-effect model to minimize bias (Cai and Fan, 2020). Otherwise, the fixed-effects model was used. The *p*-value less than 0.05 was considered statistically significant.

## 3 Results

### 3.1 Characteristics of the included studies

Upon searching the database using specific keywords, approximately 350 studies were identified. When reviewing the titles and abstracts, 264 studies were excluded. Following a full-text review, only 14 studies were included in our meta-analysis (Abhishek et al., 2015; Kumar et al., 2015; Girish et al., 2016; Kc et al., 2016; Puvvada et al., 2016; Joshi et al., 2017; Boulos and Nada, 2018; Goyal et al., 2018; Teama et al., 2020; Falahatkar et al., 2021; Gur et al., 2021; Khouni et al., 2022; Abdelaal and El-Dydamony, 2023; Sharma et al., 2023). The details of the selection procedure are shown in Supplementary Figure S1, and details of included studies and patient characteristics are shown in Table 1.

**TABLE 1:** Details of enrolled studies and patient characteristics.

Study (years)	Treatment	Age (year)	Patients	Stone size (mm)	Dose (mg)	Duration (week)
Abdelaal and El-Dydamony (2023)	Tamsulosin	38.7	50	5–10	0.4	4
	Tadalafil	41.9	50		5	
Abhishek et al. (2015)	Tamsulosin	NA	50	4–10	0.4	2
	Tadalafil	NA	50		10	
Boulos and Nada (2018)	Tamsulosin	36.54	50	<10	0.4	4
	Tadalafil	35.36	50		10	
Falahatkar et al. (2021)	Tamsulosin	37	44	<10	0.4	4
	Tadalafil	37.76	44		10	
Girish et al. (2016)	Tamsulosin	37.3	30	<10	0.4	4
	Tadalafil	33	30		5	
Goyal et al. (2018)	Tamsulosin	42.13	61	6–10	0.4	4
	Tadalafil	42.61	62		10	
Gur et al. (2021)	Tamsulosin	41	48	4–10	0.4	4
	Tadalafil	40.2	46		5	
Joshi et al. (2017)	Tamsulosin	NA	109	5–10	NA	4
	Tadalafil	NA	109		NA	
Kc et al. (2016)	Tamsulosin	31.37	41	5–10	0.4	2
	Tadalafil	32.05	44		10	
Khouni et al. (2022)	Tamsulosin	NA	42	5–10	0.4	6
	Tadalafil	NA	40		5	
Kumar et al. (2015)	Tamsulosin	36.4	90	5–10	0.4	4
	Tadalafil	37.5	90		10	
Puvvada et al. (2016)	Tamsulosin	37.53	100	5–10	0.4	4
	Tadalafil	36.34	100		10	
Sharma et al. (2023)	Tamsulosin	38.46	55	5–10	0.4	3
	Tadalafil	34.94	57		10	
Teama et al. (2020)	Tamsulosin	20–40	20	<8	0.4	3
	Tadalafil		20		5	

All the included studies were prospective randomized clinical trials comparing the efficacy and safety of tamsulosin and tadalafil in treating distal ureteral stones smaller than 10 mm in length. The risk of bias summary was estimated and presented in Supplementary Figure S2.

### 3.2 Stone expulsion

**FIGURE 1:**
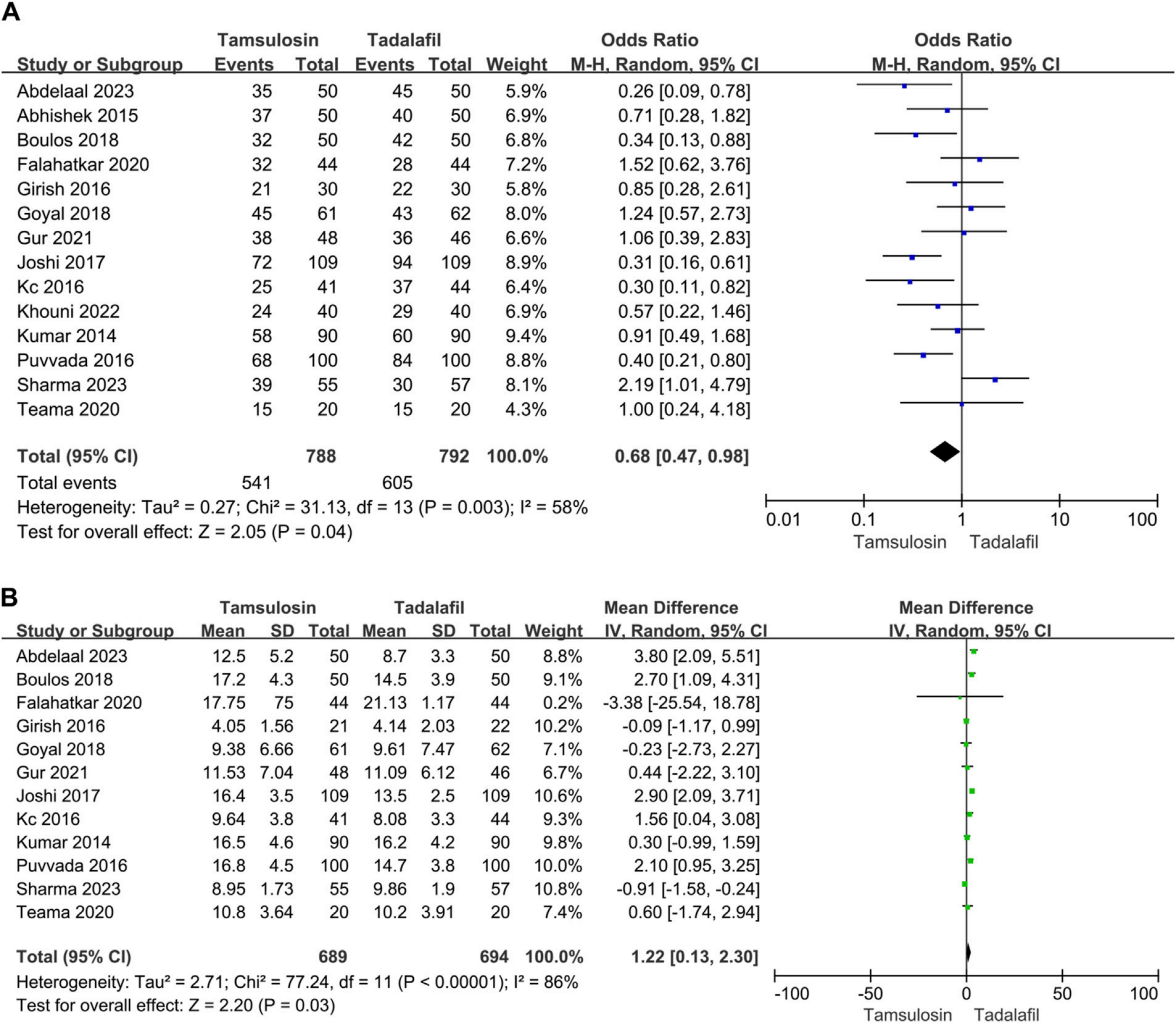
Forest plots showing the result between tadalafil and tamsulosin in **(A)** expulsion rate, **(B)** expulsion time. M–H, Mantel–Haenszel; CI, confidence interval; df, degrees of freedom.

All enrolled studies reported expulsion rates for tamsulosin and tadalafil among 1,580 patients. Significant heterogeneity was observed between the studies (I^2^ = 58%, *p* = 0.003); consequently, a random-effect model was applied to mitigate bias in the estimates. The result indicated that tadalafil was more effective than tamsulosin in improving expulsion rates [OR = 0.68, 95% CI (0.47, 0.98), *p* = 0.04] (Figure 1A). About 12 studies involving 1,393 patients reported on expulsion time. Heterogeneity was also observed in the analysis (I^2^ = 86%, *p* < 0.00001), leading to the adoption of a random-effect model for analysis, which identified that tadalafil could shorten expulsion time of distal ureteral stones compared with tamsulosin [MD = 1.22, 95% CI (0.13, 2.30), *p* = 0.03] (Figure 1B).

### 3.3 Analgesia use, pain episodes and hospital visit

**FIGURE 2:**
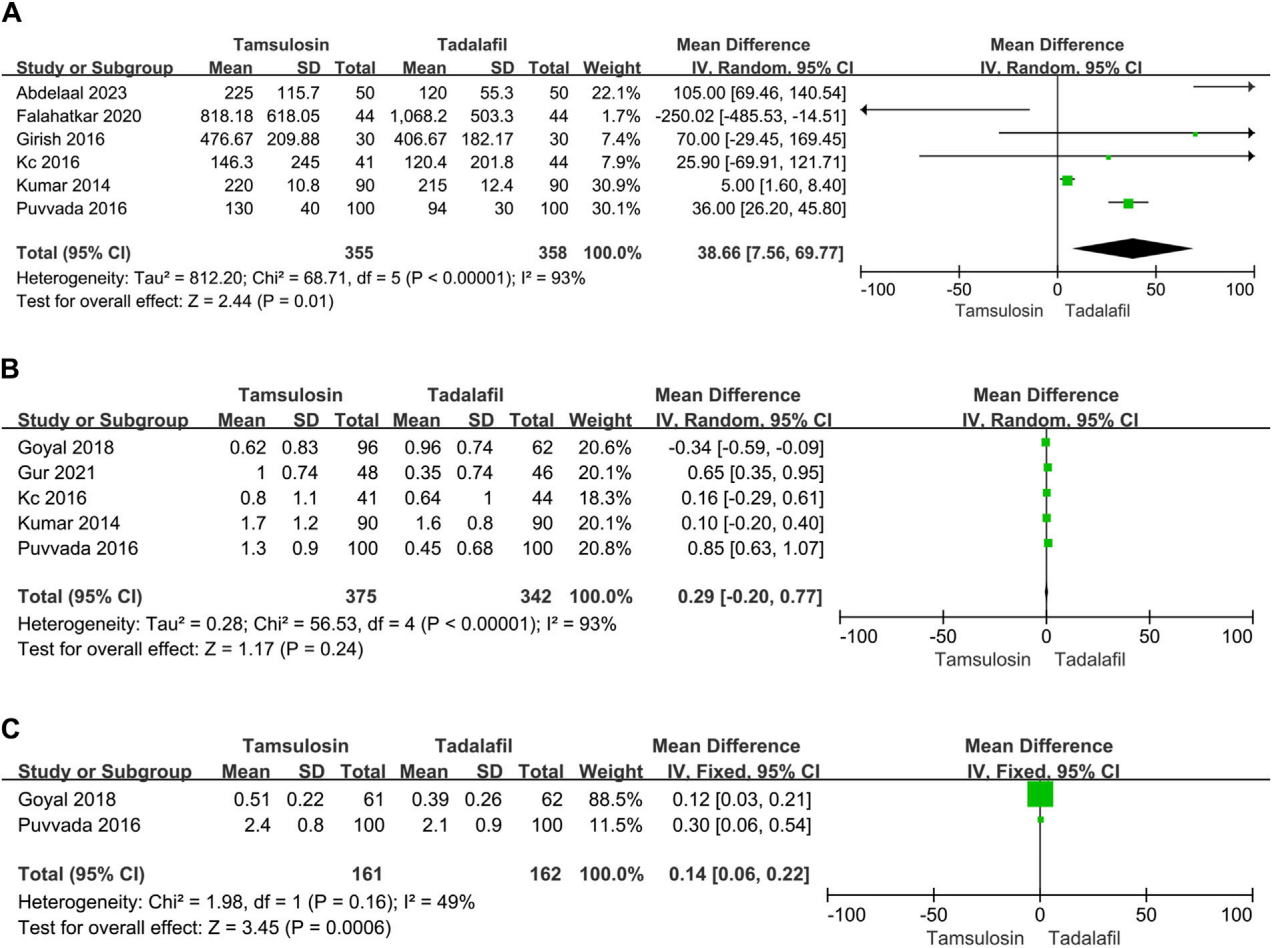
Forest plots showing the result between tadalafil and tamsulosin in **(A)** need for analgesia, **(B)** pain episodes and **(C)** hospital visit. M–H, Mantel–Haenszel; CI, confidence interval; df, degrees of freedom.

Only six studies reported analgesia usage (NSAID), encompassing 355 patients in the tamsulosin group and 358 patients in the tadalafil group. The enrolled studies showed significant heterogeneity (I^2^ = 93%, *p* < 0.00001). The random-effect model estimated that NSAID usage was lower in patients treated with tadalafil than tamsulosin [MD = 38.66, 95% CI (7.56, 69.77), *p* = 0.01] (Figure 2A). Additionally, patients treated with tadalafil were less likely to visit the hospital due to pain compared to those treated with tamsulosin [MD = 0.14, 95% CI (0.06, 0.22), *p* = 0.0006] by summarizing results of two studies (Figure 2C). However, the number of pain episodes collected from five studies did not differ between tadalafil and tamsulosin treatments [MD = 0.29, 95% CI (−0.2, 0.77), *p* = 0.24] (Figure 2B).

### 3.4 Side effects

The side effects mentioned in more than two studies were six types, including headache, abnormal ejaculation, backache, dizziness, orthostatic hypotension, and gastritis. The heterogeneity was not significant in various analyses. The fixed-effect mode was selected to conduct these analyses. Our analysis of 817 patients across 7 studies revealed that patients using tamsulosin were more likely to experience abnormal ejaculation compared to those using tadalafil [OR = 2.78, 95% CI (1.57, 4.94), *p* = 0.0005] (Supplementary Figure S3B). The incidence of other side effects did not differ for headache [OR = 0.70, 95% CI (0.47, 1.03), *p* = 0.07], backache [OR = 0.75, 95% CI (0.49, 1.16), *p* = 0.2], dizziness [OR = 0.91, 95% CI (0.58, 1.43), *p* = 0.7], orthostatic hypotension [OR = 1.35, 95% CI (0.84, 2.18), *p* = 0.22], and gastritis [OR = 0.44, 95% CI (0.16, 1.2), *p* = 0.11] (Supplementary Figure S3).

### 3.5 Subgroup analysis

According to different dosage of tadalafil (5mg and 10 mg), we also performed subgroup analysis. Approximately five studies reported treatment with tadalafil 5mg, while tadalafil 10 mg was adopted in the other eight studies. Only one study did not mention the dosage of tamsulosin and tadalafil.

#### 3.5.1 Tadalafil 5 mg

##### 3.5.1.1 Stone expulsion and analgesia use

**FIGURE 3:**
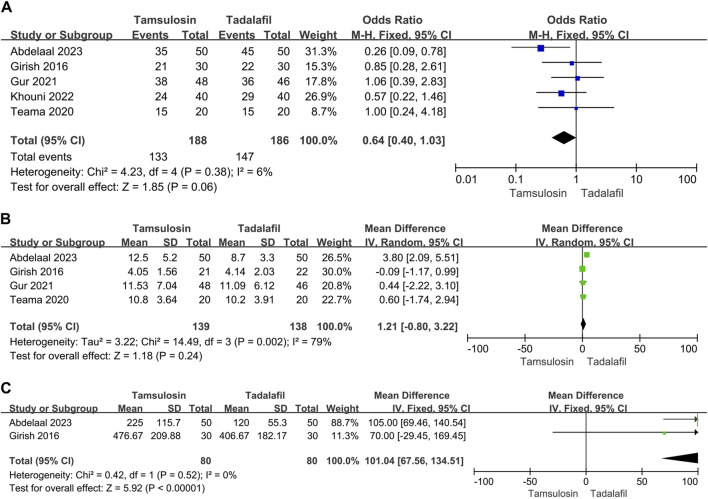
Forest plots showing the result between tadalafil 5 mg and tamsulosin in **(A)** expulsion rate, **(B)** expulsion time and **(C)** need for analgesia. M–H, Mantel–Haenszel; CI, confidence interval; df, degrees of freedom.

Five studies reported the expulsion rate, involving 374 patients, and four studies reported the expulsion time, involving 277 patients. There was significant heterogeneity in the expulsion time analysis, and the random-effect model was selected. The efficacy was the similar in expulsion rate [OR = 0.64, 95% CI (0.4, 1.03), *p* = 0.06] and expulsion time [MD = 1.21, 95% CI (−0.8, 3.22), *p* = 0.24] between tadalafil 5 mg and tamsulosin. However, analgesia usage was lower in patients receiving tadalafil 5 mg compared to those receiving tamsulosin [MD = 101.04, 95% CI (67.56, 134.51), *p* < 0.00001], although this finding was based on limited data (Figure 3).

##### 3.5.1.2 Side effects

Only two studies reported side effects, including headache, abnormal ejaculation, backache, and orthostatic hypotension. Patients receiving tadalafil 5 mg were less likely to experience abnormal ejaculation [OR = 3.08, 95% CI (1.06, 8.92), *p* = 0.04] and orthostatic hypotension [OR = 3.52, 95% CI (1.24, 10), *p* = 0.02] compared to those receiving tamsulosin (Supplementary Figure S4). But the incidence of headache [OR = 0.82, 95% CI (0.36, 1.87), *p* = 0.64] and backache [OR = 1.18, 95% CI (0.5, 2.76), *p* = 0.7] was not difference.

#### 3.5.2 Tadalafil 10 mg

##### 3.5.2.1 Stone expulsion and analgesia use, pain episodes

**FIGURE 4:**
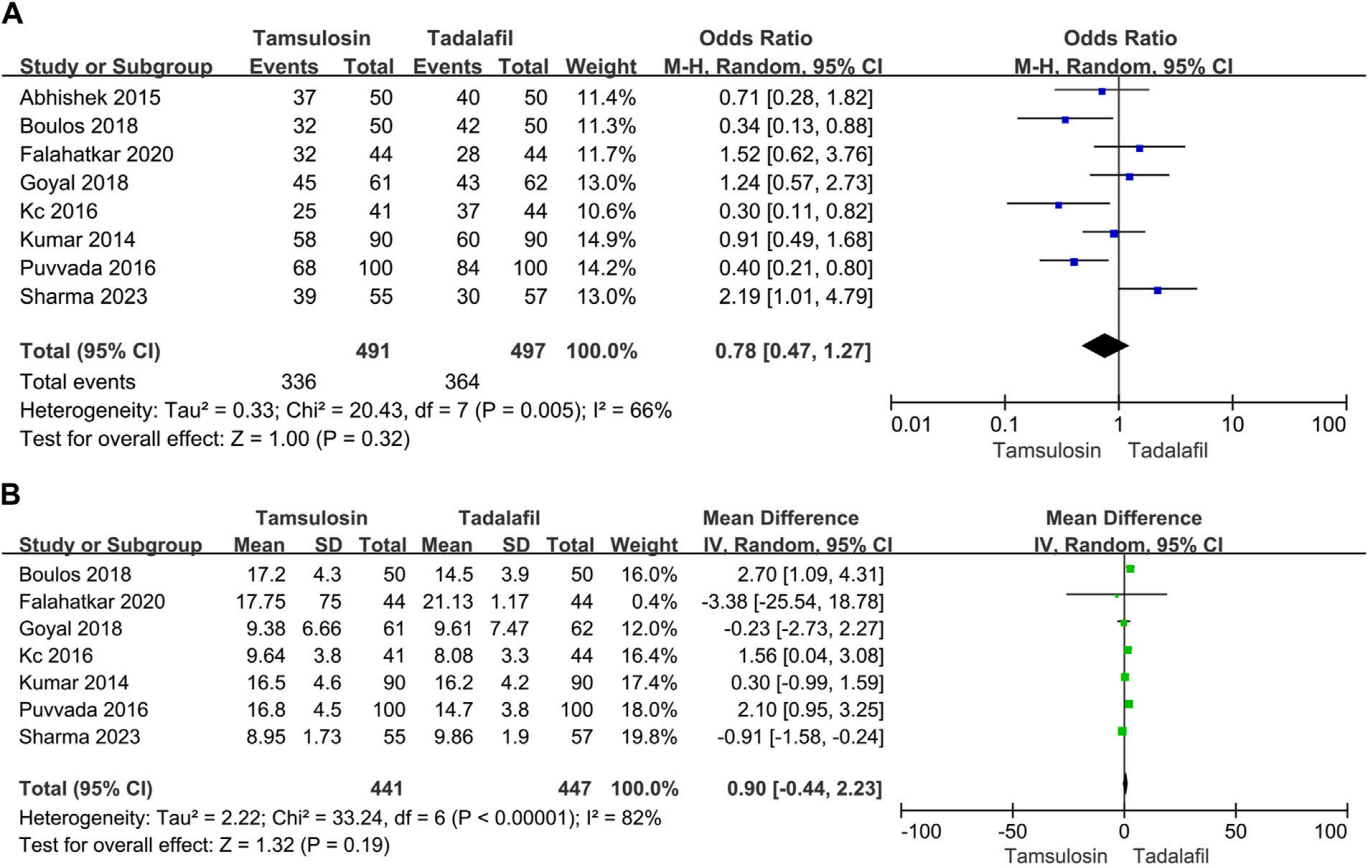
Forest plots showing the result between tadalafil 10 mg and tamsulosin in **(A)** expulsion rate, **(B)** expulsion time. M–H, Mantel–Haenszel; CI, confidence interval; df, degrees of freedom.

In eight studies, 497 patients received 10 mg tadalafil, while approximately 491 patients received tamsulosin. The estimates for expulsion rate and time showed significant heterogeneity (rate: I2 = 66%, *p* = 0.005; time: I2 = 82%, *p* < 0.00001). The random-effect model revealed that tadalafil did not exhibit better efficiency in either expulsion rate [OR = 0.78, 95% CI (0.47, 1.27), *p* = 0.32] or expulsion time [MD = 0.9, 95% CI (−0.44, 2.23), *p* = 0.19] compared to tamsulosin (Figure 4).

Heterogeneity tests for analgesia use and pain episodes were also significant. The results indicated no statistically significant differences when comparing analgesia use [MD = 16.44, 95% CI (−13.4, 46.27), *p* = 0.28] and pain episodes [MD = 0.2, 95% CI (−0.39, 0.79), *p* = 0.52] using the random-effect model (Supplementary Figures S5A, S5B).

##### 3.5.2.2 Side effects

In terms of safety, only abnormal ejaculation occurred more frequently with tamsulosin compared to tadalafil 10 mg [OR = 2.67, 95% CI (1.35, 5.68), *p* = 0.005]. The incidence of other side effects, including headache [OR = 0.67, 95% CI (0.43, 1.04), *p* = 0.07], backache [OR = 0.64, 95% CI (0.38, 1.07), *p* = 0.09], dizziness [OR = 0.8, 95% CI (0.5, 1.3), *p* = 0.37] and orthostatic hypotension [OR = 0.98, 95% CI (0.56, 1.71), *p* = 0.94], did not differ significantly (Supplementary Figures S5C–S5G).

## 4 Discussion

Urolithiasis, a common urological disease, significantly impacts quality of life (Shokeir et al., 2016). Approximately 20% of urolithiasis cases involve ureteral calculi, which typically present with acute onset and severe symptoms, with 68% occurring in the distal ureter (Bai et al., 2017). The MET, endorsed by American and European Guidelines for treating distal ureteral stones less than 10 mm in size, includes alpha adrenoreceptor antagonists and calcium channel inhibitors, providing a safe and cost-effective option.

Several studies have reported the efficacy and safety of tamsulosin in treating distal ureteral calculi by suppressing basal smooth muscle tone (Yilmaz et al., 2005; Malo et al., 2014). With the development of medical therapy, PDE-5Is were developed initially to treat sexual dysfunction and lower urinary tract symptoms (McMahon, 2019). They were also used in treating distal ureteral stones by inducing ureteric relaxation through the smooth muscle nitric oxide/cyclic guanosine monophosphate signaling pathway (Zhou et al., 2019). A previous comprehensive meta-analysis reported higher efficacy in expulsion rate and time, with comparable safety between tadalafil and tamsulosin, based on four studies (Bai et al., 2017).

In this meta-analysis, we enrolled 1,580 patients from 14 studies, with 788 patients receiving tamsulosin and 792 receiving tadalafil. Our results showed that tadalafil significantly increased the expulsion rate, shortened the expulsion time, and decrease analgesia use compared to tamsulosin. We also identified for the first time that tadalafil reduced hospital visits due to severe pain in treatment of distal ureteral calculi. Based on our analysis, we indicated that tadalafil had similar safety profiles to tamsulosin in terms of backache, dizziness, orthostatic hypotension, and gastritis. However, we found that tamsulosin was more likely than tadalafil to cause abnormal ejaculation, possibly due to neurogenic relaxation of the prostate and bladder neck (Cicione et al., 2023).

Recently, there has been a growing focus in studies on the use of a low dose of 5 mg tadalafil for treating erectile dysfunction, premature ejaculation, and benign prostatic hyperplasia (Karabakan et al., 2017; Abou Faddan et al., 2022). In patients with lower urinary tract symptoms secondary to benign prostatic hyperplasia, 5 mg tadalafil was found to be the best tolerated treatment with long-lasting efficacy (Donatucci et al., 2011). Subgroup analysis was also conducted based on tadalafil doses 5 mg and 10 mg.

We firstly summarized studies to compare the efficacy and safety of tadalafil 5 mg and tamsulosin in treatment of distal ureteral stone less than 10 mm in size. However, the results showed similar efficacy in terms of expulsion rate and time. The patients receiving tadalafil showed a reduction in analgesic use. Moreover, the incidence of orthostatic hypotension and abnormal ejaculation was lower with tadalafil 5 mg. Both groups tolerated headache and backache treatments well. As for the comparison between tadalafil 10 mg and tamsulosin, eight studies involving 988 patients were included in this analysis. There was no difference in efficacy for primary outcomes, including expulsion rate and expulsion time. In terms of safety, abnormal ejaculation occurred more frequently in patients treated with tamsulosin. There were no differences between the two groups in the other side effects, including headache, orthostatic hypotension, dizziness and backache.

In this meta-analysis, we identified that tadalafil is more efficient than tamsulosin with favorable safety profiles in treating distal ureteral calculi smaller than 10mm, based on a larger scale of studies. However, the ideal dosage of tadalafil for treating distal ureteral calculi remains uncertain, and further researches is needed to confirm this. Readers must understand the limitations of this meta-analysis. Firstly, the quality of included studies was heterogeneous, particularly in study design, allocation concealment, and blinding, which could introduce information bias and selection bias. Therefore, explanations of the results should be approached cautiously. Secondly, the scale of enrolled studies was limited, especially in subgroup analysis. We will continue to search for the latest researches to comprehensively address this limitation in the future. Finally, additional high-quality clinical researches are needed to confirm these results.

## 5 Conclusion

Compared with tamsulosin, tadalafil demonstrates a higher expulsion rate and shorter expulsion time for patients with distal ureteral stones less than 10 mm and a favorable safety profile, which warrants further investigation in additional high-quality studies.

## Data Availability

The original contributions presented in the study are included in the article/Supplementary Material, further inquiries can be directed to the corresponding authors.
